# Unusual presentation of a skull base mass lesion in sarcoidosis mimicking malignant neoplasm: a case report

**DOI:** 10.1186/s12883-018-1076-6

**Published:** 2018-05-29

**Authors:** Katsunori Shijo, Nobuhiro Moro, Mari Sasano, Mitsuru Watanabe, Hiroshi Yagasaki, Shori Takahashi, Taku Homma, Atsuo Yoshino

**Affiliations:** 1 Department of Neurological Surgery, Nihon University School of Medicine, 30-1 Oyaguchi-Kamimachi, Itabashi-ku, Tokyo, 173-8610 Japan; 2 Department of Pediatrics and Child Health, Nihon University School of Medicine, Tokyo, Japan; 3 Division of Human Pathology, Department of Pathology and Microbiology, Nihon University School of Medicine, Tokyo, Japan

**Keywords:** Neurosarcoidosis, Pediatric, Sarcoidosis, Intracranial mass lesion, MRI

## Abstract

**Background:**

Sarcoidosis is a multi-organ disease of unknown etiology characterised by the presence of epithelioid granulomas, without caseous necrosis. Systemic sarcoidosis is rare among children, while neurosarcoidosis in children is even rarer whether it is systemic or not.

**Case presentation:**

We described the case of a 12-year-old boy who presented with monocular vision loss accompanied by unusual MRI features of an extensive meningeal infiltrating mass lesion. The patient underwent surgical resection (biopsy) via a frontotemporal craniotomy to establish a definitive diagnosis based on the histopathology, since neurosarcoidosis remains a very difficult diagnosis to establish from neuroradiogenic imagings. Based on the histopathology of the resected mass lesion, neurosarcoidosis was diagnosed. On follow-up after 3 months of steroid therapy, the patient displayed a good response on the imaging studies. MRI revealed that the preexisting mass lesion had regressed extremely. We also conducted a small literature review on imaging studies, manifestations, appropriate treatments, etc., in particular neurosarcoidosis including children.

**Conclusion:**

Although extremely rare, neurosarcoidosis, even in children, should be considered in the differential diagnosis of skull base mass lesions to avoid unnecessary aggressive surgery and delay in treatment, since surgery may have little role in the treatment of sarcoidosis.

## Background

Sarcoidosis is an idiopathic granulomatous disease which commonly involves the lungs, skin, and eyes, with simultaneous or metachronous features [1, 2]. Sarcoidosis involving the central nervous system (CNS; neurosarcoidosis) occurs in approximately 5-15% of adults with systemic sarcoidosis [3, 4]. Even fewer cases of sarcoidosis have been reported in children, especially cases affecting the CNS [5]. Baumann and Robertson [6] reviewed pediatric neurosarcoidosis and concluded that childhood neurosarcoidosis differs in its presenting signs and symptoms from neurosarcoidosis in adults; children are more likely to have seizures, less likely to have cranial nerve palsies, and perhaps more likely to have a space-occupying lesion. More recently, Rao et al. [4] reviewed neurosarcoidosis in pediatric patients. They found that only 53 examples of neurosarcoidosis had been reported in the pediatric population, with nine of these cases being isolated neurosarcoidosis. The most common manifestations included cranial neuropathy (21%), papilledema or optic neuritis (15%), seizures (24.5%), and hypothalamic dysfunction (17%), with the latter two conditions being more likely in younger children.

We described here the rare clinical and radiological manifestations of 12-year-old boy with monocular vision loss who was initially thought to have a skull base tumor, but was subsequently confirmed to have neurosarcoidosis with systemic sarcoidosis. In addition, we present a small literature review of neurosarcoidosis, especially in pediatric patients.

## Case presentation

### History and examinations

A 12-year-old boy was introduced to our institution because he displayed visual disturbance and was described as having an intracranial mass lesion by another hospital. His family history was unremarkable. His past history included a right neck mass lesion: this had expanded to 40 mm in 1 month and was histologically suspected to be associated with sarcoidosis by another hospital 2 years ago. Subsequently, he was followed up at that hospital, but there was no obvious abnormality until 3 months ago when visual disturbance was identified at an annual school medical checkup.

His consciousness level was clear and neurological examinations demonstrated no abnormalities except for the ophthalomological problem. Neurological examinations, including the I and III – XII cranial nerves, detected no abnormality. Ophthalomological evaluations revealed concentric contraction of the visual field and diminished visual acuity of the right eye. His best corrected visual acuity was 0.2/20 (n.c) in the right eye and 4/20 (1.2pxS-1.5D) in the left eye. The pupils were semidilated and the pupillary light reflex was sluggish on the right side, while a normal size and normal reaction were evident on the left side. In a fundus examination, his right optic disc was found to be pale.

Routine laboratory tests including renal function and hormonal tests demonstrated no obvious abnormalities. As a marker for sarcoidosis, his serum angiotensin-converting enzyme (ACE) was slightly increased at 22.5 U/L (8.3 to 21.5 U/L). His chest X-ray performed on admission showed no obvious abnormalities, but a subsequent chest computed tomography (CT) scan revealed small scattered pulmonary nodules in the bilateral lungs.

**Fig. 1:**
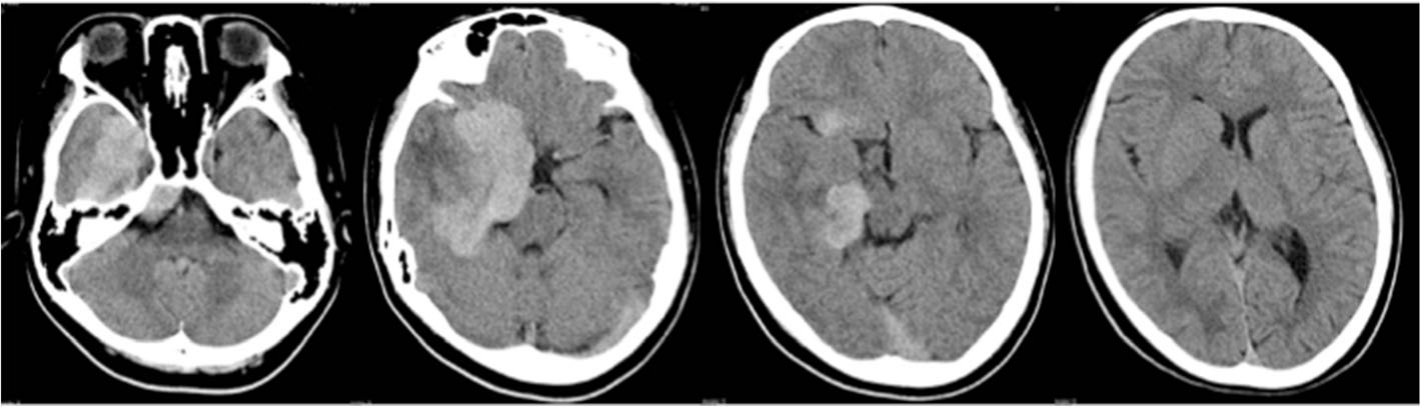
Preoperative plain CT scan of the head showing a slightly high density huge skull base mass lesion on the right side

**Fig. 2:**
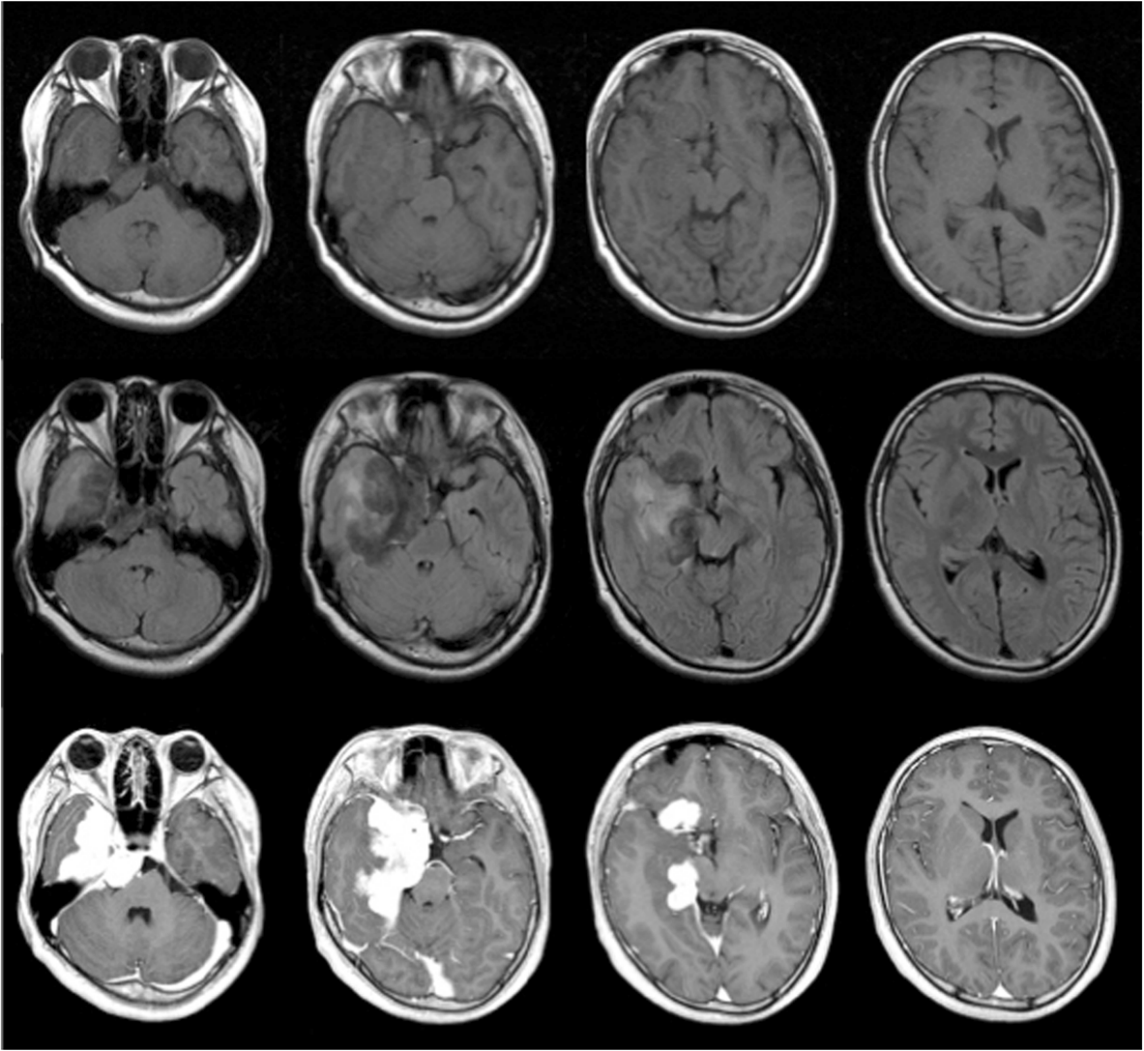
Preoperative axial MRI of the brain. T1-weighted (upper row) and fluid-attenuated inversion recovery (FLAIR; middle row) images demonstrated iso-intensity and a low-intensity mass lesion with edema in the right skull base, respectively. T1-weighted images after administration of gadolinium (lower row) revealed an homogeneous enhanced leaf-shaped huge mass lesion with pachymeningeal and leptomeningeal enhancement involving the tentorium

**Fig. 3:**
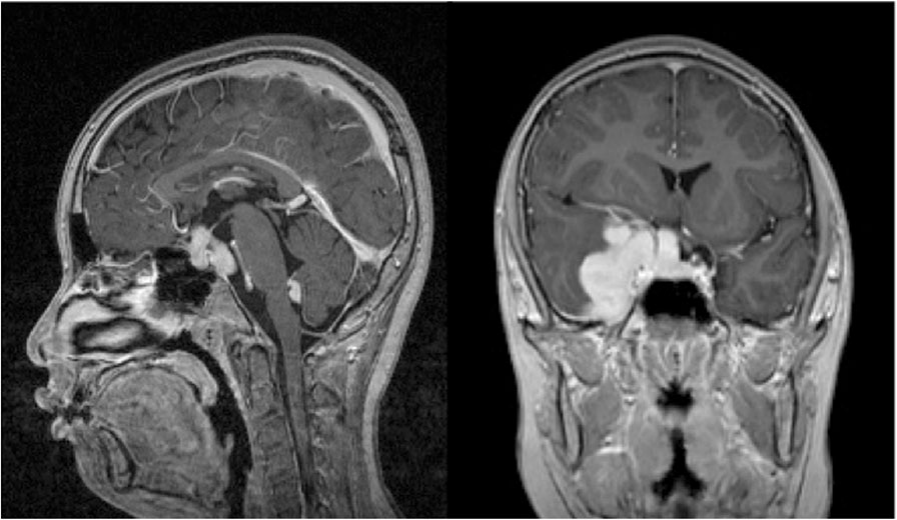
Sagittal T1-weighted MRI with gadolinium (left) demonstrating a small enhanced mass of the fourth ventricle and an homogeneous enhanced mass in the pituitary and infundibulum. Further, coronal T1-weighted MRI with gadolinium (right) revealed extensive involvement of the right cavernous sinus and the presence of dural enhancement resembling a dural tail sign in the right middle cranial fossa

A CT scan and magnetic resonance imaging (MRI) of the head demonstrated that a leaf-shaped extra-axial mass of about 73 mm in length was present around the right cavernous sinus, straddling the sella turcica, frontal, middle, and posterior cranial fossa, with dural thickness to the tentorium cerebrii (Figs. 1, 2, 3). The greater part of this lesion showed iso signal intensity on T1 weighted images and low signal intensity on T2 weighted images, while homogeneous enhancement was evident after contrast medium administration. Perilesional edema was not obviously found in the adjacent brain except the right temporal lobe. Furthermore, an enhanced nodular shadow of 12 mm in size was detected in the lower part of the fourth ventricle. Spinal MRI revealed no abnormality throughout the spinal cord. Right internal carotid artery and external carotid artery angiographies showed week staining where the mass was located on the MRI, although it was difficult to locate the origin of these feeding arteries. The above imaging findings indicated an extra-axial large tumor of the right skull base suggesting meningioma, solitary fibrous tumor, etc.

### Surgery

The patient underwent surgical resection (biopsy) via a frontotemporal craniotomy to establish a definitive diagnosis based on the histopathology, since neurosarcoidosis remains a very difficult diagnosis to establish from neuroradiogenic imagings.

The intraoperative findings indicated that the tumor was hard, and visually and tactually resembled like a meningioma. The lesion was found immediately after opening the sylvian fissure, and was easy to separate from the surrounding brain parenchyma in the frontal cortex, but had no distinct boundaries in the temporal cortex. When reaching deeply into the sylvian fissure, the internal cerebral artery was seen to be involved within the lesion. The immediate pathology revealed that the specimen consisted of a proliferation of short spindle cells without necrosis, with multinucleated giant cells. Surgical exploration was therefore terminated at this stage.

### Histopathological examinations

**Fig. 4:**
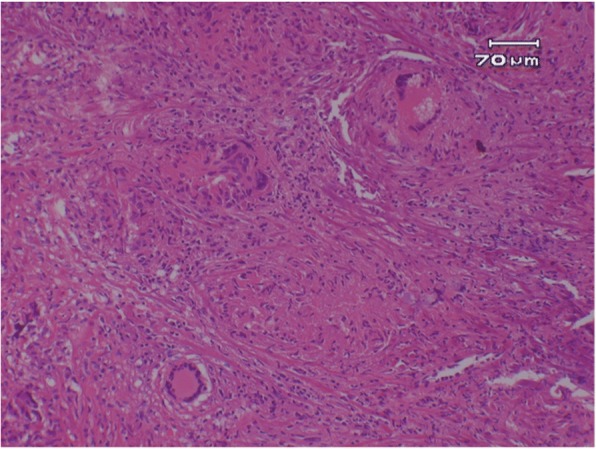
Biopsy specimen showing noncaseating epithelioid cell granulomas with multinucleated giant cells (hematoxylin and eosin stain: original magnification × 100)

The definitive histopathology of the resected mass lesion showed epithelioid cell granulomas and Langhans type multinucleated giant cells that were rich in dense fibrous tissue with lymphocytes and plasma cells. Necrosis was not clear in the granulomas observed in the specimen (Fig. 4). Grocott-positive fungi and Ziehl-Neelsen staining positive anti-acid bacteria were not clearly found. Non-caseating granulomatous inflammation consistent with neurosarcoidosis was diagnosed.

### Post-operative course

Based on the histopathology of the resected mass lesion, neurosarcoidosis was diagnosed. Although steroid therapy was planned, the start was delayed due to epidural abscess of the surgical wound, which required a surgical procedure and antibacterial therapy. Three months after the biopsy, the patient was started on steroid therapy (prednisolone at 1.0 mg/kg body weight per day; reduction of the prednisolone was made at 5 mg every 4 weeks). Fortunately, during this delayed time, his neurological examinations did not change and brain MRI also indicated no local growth of the lesion where the mass lesion was left.

**Fig. 5:**
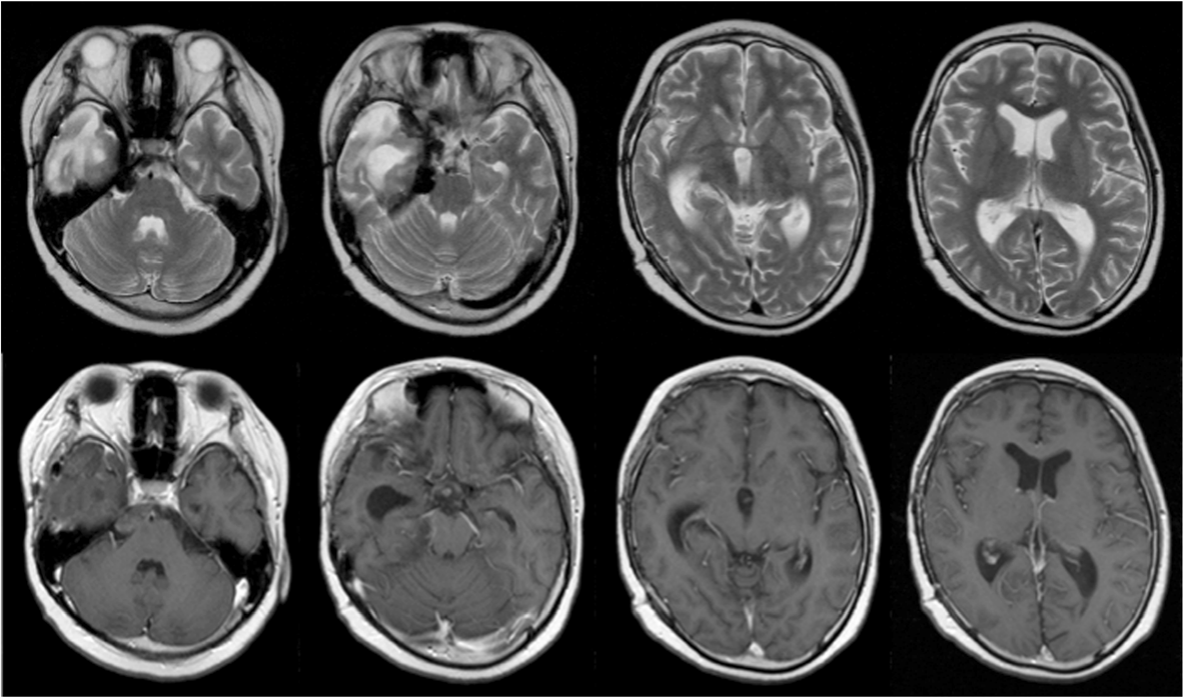
T2-weighted MRI (upper row) and T1-weighted MRI with gadolinium (lower row) at 3 months after medical therapy demonstrating that the mass, with iso signal intensity on T1-weighted images and extremely low signal intensity on T2-weighted images, had involuted considerably with a reduction in enhancement

On follow-up after 3 months of steroid therapy, the patient displayed a good response on the imaging studies. MRI revealed that the preexisting mass lesion had regressed extremely. The enhanced small nodular shadow in the lower part of the fourth ventricle had disappeared. Only around the right cavernous sinus, did the findings show a residual small mass of iso signal intensity on T1-weighted images and an extremely low signal intensity on T2-weighted images with no enhancement effect after contrast medium administration (Fig. 5).

When the prednisolone reaches 25 mg/day, additional treatment using methotrexate in combination with prednisolone is planned for the patient.

## Discussion and conclusions

Intracranial pseudotumors are uncommon, but it is important to recognize such lesions. They may occur in inflammatory diseases (systemic diseases, vasculitis, and demyelinating diseases), infectious diseases, vascular diseases, and radionecrosis [7]. Among such conditions, sarcoidosis is a multi-organ disease of unknown etiology, but is thought to be associated with an abnormal immune response, and develops from young people to elderly people with a peak in the fourth decade [1, 8]. The clinical symptoms at the time of onset are diverse, and the subsequent clinical course is also diverse. Sarcoidosis most commonly affects the lungs and thoracic lymph nodes; however, virtually any organ system can be involved including the eyes, skin, kidney, heart, and CNS [2, 3, 8]. Sarcoidosis may involve any of the intracranial regions including the supratentorial and infratentorial compartments as well as leptomeningeal and intraparenchymal areas, with solitary or multiple lesions, and such involvement gives rise to a broad variety clinical manifestations [1, 3, 9, 10]. Thus, a significant number of patients with neurosarcoidosis may have asymptomatic lesions or be misdiagnosed [8]. It has been reported that ^18^F-FDG PET/CT is a valuable tool for the diagnostic evaluation of patients suspected of sarcoidosis and is also important for other major diagnostic discrimination, including lymphoma and metastasis [11].

Although imaging studies are nonspecific, MRI is a very sensitive diagnostic tool for the finding and detection of intracranial lesions due to neurosarcoidosis [3]. Previous reports have regarded mass lesions as rare in pediatric neurosarcoidosis [12], however Baumann and Robertson reported 24% had mass lesions on imaging and it is unclear whether children have different patterns from adults [6]. The lesions are frequently reported to be isointensive on T1-weighted images and hypointensive masses on T2-weighted images, and to be uniformly enhanced after contrast medium injection [3]. Other common findings in pediatric neurosarcoidosis include periventricular high signal lesions on T2-weighted images and leptomeningeal enhancement on T1-weighted images with contrast [6]. Wiśniewski et al. [13] pointed out the relationship between the observation of extremely low signal on T2-weighted images and noncaseating granuloma in their neurosarcoidosis case. In our patient, the low signals on T2-weighted images further advanced after steroid therapy, which may indicate an inactive lesion, although we need to continue observation of the future course.

One of the typical images of neurosarcoidosis is leptomeningeal involvement which usually appears as a thickening and diffuse and/or focal enhancement [1, 4, 10]. Such an image may be readily misidentified for diseases such as glioma, meningioma, metastatic tumor, demyelinating disease, vasculitis, and other granulomatous diseases including tuberculosis, Wegener’s granulomatosis, etc. [3, 13]. Thus, even in children, we need to include the possibility of neurosarcoidosis in the differential diagnosis of intracranial lesions [3, 13, 14].

It has been reported that cranial neuropathies, which may involve one or more nerves and occur unilateral or bilaterally [8], are seen in about 60% of adult patients with neurosarcoidosis [4, 8]. Among them, facial nerve palsy is suggested to be the most commonly encountered neurologic complication [4, 6], although it could include cases related to parotitis. Carlson et al. [8], who reviewed the cranial base manifestations of neurosarcoidosis, found that the optic, trigeminal, and facial nerve(s) represented the most frequently involved neuropathies, in that order. On the other hand, Rao et al. [4], who reviewed neurosarcoidosis in pediatric patients, indicated that the prepubertal aged group most commonly presented with seizures, while postpubertal children presented similarly to adults and were more likely to have cranial neuropathy; facial nerve palsy was the most common condition, followed by acoustic neuropathy and optic nerve involvement. The mechanism of such neuropathy may not only result from nerve granuloma, but may also be due to increased intracranial pressure or granulomatous basal meningitis [4, 8].

Several studies have indicated that increased serum ACE levels may be observed in sarcoidosis, but not in all cases [6]. Furthermore, elevated ACE levels are not specific for sarcoidosis because other diseases have also revealed elevated serum ACE levels including tuberculosis, lung cancer, Hodgkin lymphoma, liver cirrhosis, etc. [4]. Thus, an elevated serum ACE does not provide a definitive diagnosis [4]. It is also controversial as to whether the level of ACE in the cerebral spinal fluid is useful for the diagnosis of neurosarcoidosis [1]. However, sequential serum ACE levels have been employed as a marker of disease progression in sarcoidosis [4].

Although criteria for the pediatric diagnosis of neurosarcoidosis have not yet been established, the most widely used criteria are those developed for adults by Zajicek et al. [4, 15]. Definitive diagnosis is based on histological evidence, which can prove non-caseating granulomas and multinucleated giant cells with surrounding lymphocytes [9], and must exclude differential diagnoses such as tuberculosis, berryliosis, and Sjögren’s syndrome which can elicit a similar histological picture. [4, 5]. It is important and necessary therefore to establish a histopathological diagnosis as far as possible, and to carry out an exclusion diagnosis adequately. Dural or leptomeningeal involvements with sarcoidosis are preferred for biopsies, because such sites require less invasive procedures as compared to brain or spinal cord biopsies [1].

The most appropriate treatment for pediatric neurosarcoidosis is remains uncertain. There is no evidence of clinical benefit from surgical removal for neurosarcoidosis [9], and the role of surgical removal may be limited to diagnostic biopsy rather than therapeutic resection. In general, corticosteroids are the most commonly used first line agents and are often effective [5, 6]. For adult patients, other agents such as immunomodulating and/or cytotoxic agents, including azathioprine, cyclophosphamide, cyclosporine A, methotrexate, etc. have been employed in combination with corticosteroids to provide better outcomes, in either severe cases, steroid-resistant cases, recurrent cases, or cases in which steroids cause side effects [1, 5, 6, 10]. 25% of the patients are refractory to steroid treatment and 20 - 40% of such refractory patients are not responsive to immunomodulating agents [10]. Although it has been indicated that adults with seizures and other intracranial lesions could be less responsive to treatment [6], our patient displayed a good response to high-dose prednisolone with slow taper treatment for 3 months including significant evidence of radiological and clinical improvement. This may be because pediatric patients can show improved lesions with corticosteroids alone [4]. The efficacy of immunomodulating and/or cytotoxic agents in children with neurosarcoidosis has not yet been fully elucidated [4]. We clearly need to undertake carefull follow-up of our patient’s future course.

## Abbreviations

ACE: Angiotensin-converting enzyme
CNS: Central nervous system
CT: Computed tomography
MRI: Magnetic resonance imaging
